# Prosthetic joint infection caused by *Pasteurella multocida*: a case series and review of literature

**DOI:** 10.1186/s12879-016-1763-0

**Published:** 2016-08-20

**Authors:** Estelle Honnorat, Piseth Seng, Hélène Savini, Pierre-Olivier Pinelli, Fabrice Simon, Andreas Stein

**Affiliations:** 1 Centre de Référence des Infections Ostéo-Articulaires (CRIOA), Interrégional Sud-Méditerranée, Service des Maladies Infectieuses, Hôpital de la Conception, 147, boulevard Baille, Marseille, France; 2 Service des Maladies Infectieuses, Hôpital de la Conception, 147, boulevard Baille, 13005 Marseille, France; 3 Aix Marseille Univ, INSERM 1095, CNRS 7278, IRD 198, URMITE, Marseille, France; 4 Service de Pathologie Infectieuse et Tropicale, Hôpital Instruction des Armées Laveran, 13013 Marseille, France

**Keywords:** *Pasteurella multocida*, Arthroplasty, Prosthetic joint infection, Zoonosis, Infection, Bacteria, Human

## Abstract

**Background:**

*Pasteurella multocida* is a well-recognized zoonotic agent following dog or cat bites or scratches. Nevertheless, prosthetic joint infection caused by *P. multocida* are rarely reported.

**Method:**

We report here a series of six cases of prosthetic joint infection caused by *P. multocida* managed at a referral centre for the treatment of bone and joint infection in southern France. We also reviewed the 26 cases reported in literature.

**Results:**

The mean age of our cases was 74 years [±8.2, range 63–85]. In majority of our cases (5 cases) were associated with knee prostheses and one case with a hip prosthesis. Most of cases occurred after cat or dog scratches or licks or contact. Diagnoses of prosthetic joint infection caused by *P. multocida* were made by positive cultures of surgical biopsies or needle aspiration. Mean time delay between prosthetic joint implantation and infection onset was 7.6 years (±5.12 years, range 2–17). Local inflammation, which occurred in all six cases, was the most frequent clinical symptom, followed by pain in five cases, fever and swollen joints in four cases, and a fistula with purulent discharge inside the wound in two cases. The mean time of antibiotic therapy was 8 months. Surgical treatment with prosthesis removal was performed in three cases. Six of our cases were in remission without apparent relapse at 3 years after end of treatment.

**Conclusion:**

Prosthetic joint infections caused by *P. multocida* usually occur after animal scratches or bites, but can occasionally occur after a short animal lick. These infections are usually resulting from a contiguous infection and localized in the knee. An early antibiotic therapy after surgical debridement could avoid prosthetic withdrawal, notably in elderly patients. Patients with prosthetic joints should be warned that animals are potential sources of serious infection and urgent medical advice should be sought if they are bitten or scratched.

## Background

The number of prosthetic orthopaedic implants has increased, and the frequency of infections that can be attributed to these prostheses is a real public health problem [1]. Prosthetic joint infections typically result from infections with aerobic bacteria such as Staphylococci, Streptococci*,* Enterococci and Gram-negative bacilli [1].

Zoonotic prosthetic joint infections have been reported in previous studies such as prosthetic joint infection caused by *Brucella* sp. [2], *Salmonella sp*. [3], and *Campylobacter sp*. [4]. A recent case of hip prosthetic infection due to *Streptococcus suis* has been reported in a 74-year-old male American farmer with a history of non-Hodgkin’s lymphoma [5].

*Pasteurella multocida* is a Gram-negative nonmotile coccobacillus found worldwide. It can be found in the nasopharynx or gastrointestinal tract of wild animals, cats and dogs [6]. Human infections with *P. multocida* are most often the result of direct tissue inoculation and usually take the form of an acute local cellulitis, tenosynovitis or osteomyelitis. Septicaemia, meningitis, peritonitis and pneumonia have also been reported [7].

Prosthetic joint infection due to *P. multocida* is rare, as only 26 cases have been reported in the literature to date. These prosthetic joint infections due to *P. multocida* were associated with the same comorbidities as for other prosthetic joint infections [8] and precession by a cat or dog bite, scratching or licking distal to the affected joint [9]. The aim of this study was to review all the cases of prosthetic joint infection caused by *P. multocida* among the cases of prosthetic joint infection managed in a referral centre for the treatment of bone and joint infections (CRIOA) in southern France.

## Methods

### Study population

We retrospectively reviewed all cases of prosthetic joint infection caused by *P. multocida* among the 4686 cases of prosthetic joint infection in 14,200 patients (inpatients and outpatients >18 years) managed for bone and joint infection from January 1993 to December 2013. This study was approved by the institutional research ethics board and a written informed consent was signed by each patient. All cases were managed at the inter-regional referral centre for the treatment of bone and joint infection in southern France, grouping together four University Hospitals and a military teaching hospital with a total of 4000 beds in Marseille, France, where a local population of approximately 852,516 was recorded in January 2012.

All episodes of prosthetic joint infection caused by *P. multocida* were diagnosed based on past medical history with clinical evidence of infection using biological and/or radiological compliant data, with at least one positive culture of *P. multocida* identified from ≥ 2 deep samples based on a surgical procedure that excluded bacterial contamination. Infections involving a prosthetic joint were classified according the time of onset after implantation: early infection within a month or chronic infections after 1 month [10]. We recorded the medical history, assessing factors such as the demographic characteristics of patients, and risk factors associated with *Pasteurella* prosthetic joint infection, including medical history of animal bites or scratches, cancer, haematological malignancy, systemic or local corticosteroid treatment, diabetes mellitus and alcoholism. We also recorded the location of the *Pasteurella* prosthetic joint infection. We individually reviewed the antibiotic treatment and/or surgical treatment approach used. The clinical outcome was evaluated at 1, 3, 6, 12 and 24 months after the end of antibiotic treatment.

### Specimen collection and microbiological analysis

Deep samples obtained by surgical procedures, i.e., joint fluids, crushed tissue or bone biopsies, were inoculated on 5 % sheep blood, chocolate, Mueller-Hinton, trypticase soy and MacConkey agar plates (BioMérieux, France) and incubated at 37 °C in a 5 % CO2 atmosphere and in an anaerobic atmosphere for 10 days. For mycobacterial culture we inoculated the samples in the MGIT tubes (Becton Dickinson, Pont-De-Claix, France) or on a home-made 5 % sheep blood agar (BioMérieux, La Balme-les-Grottes, France) for 2–45 days at 32 °C or 37 °C as previously described [11, 12]. Pure bacterial cultures, obtained by picking isolated colonies, were identified with semi-automated Gram staining (Aerospray Wiescor, Elitech), catalase and oxidase activity tests, and the Vitek 2 system (BioMérieux, Marcy l’Etoile, France). The antibiotic susceptibility of *P. multocida* isolates were determined and interpreted according to the recommendations of the French Society for Microbiology (http://www.sfm-microbiologie.org/UserFiles/files/casfm/CASFM_EUCAST_V1_0_2014.pdf).

## Results

We have identified six cases of prosthetic joint infection caused by *P. multocida* among the 4686 cases of prosthetic joint infection managed in our centres over the last 20 years. The mean age of our cases was 74 years [±8.2, range 63–85]. A medical history of cat scratches was identified in two cases, dog licks on surgical wound in few days before the beginning of symptoms in two cases and close-contact with cats or dogs in two cases. In two cases occurred after dog lick, culture from the dog’s mouth cavities also yielded *P. multocida*. Two of our cases had diabetic mellitus and there was one case of class III obesity (body mass index at 55). One of our cases developed breast cancer 4 years after the prosthetic joint infection.

**Fig. 1:**
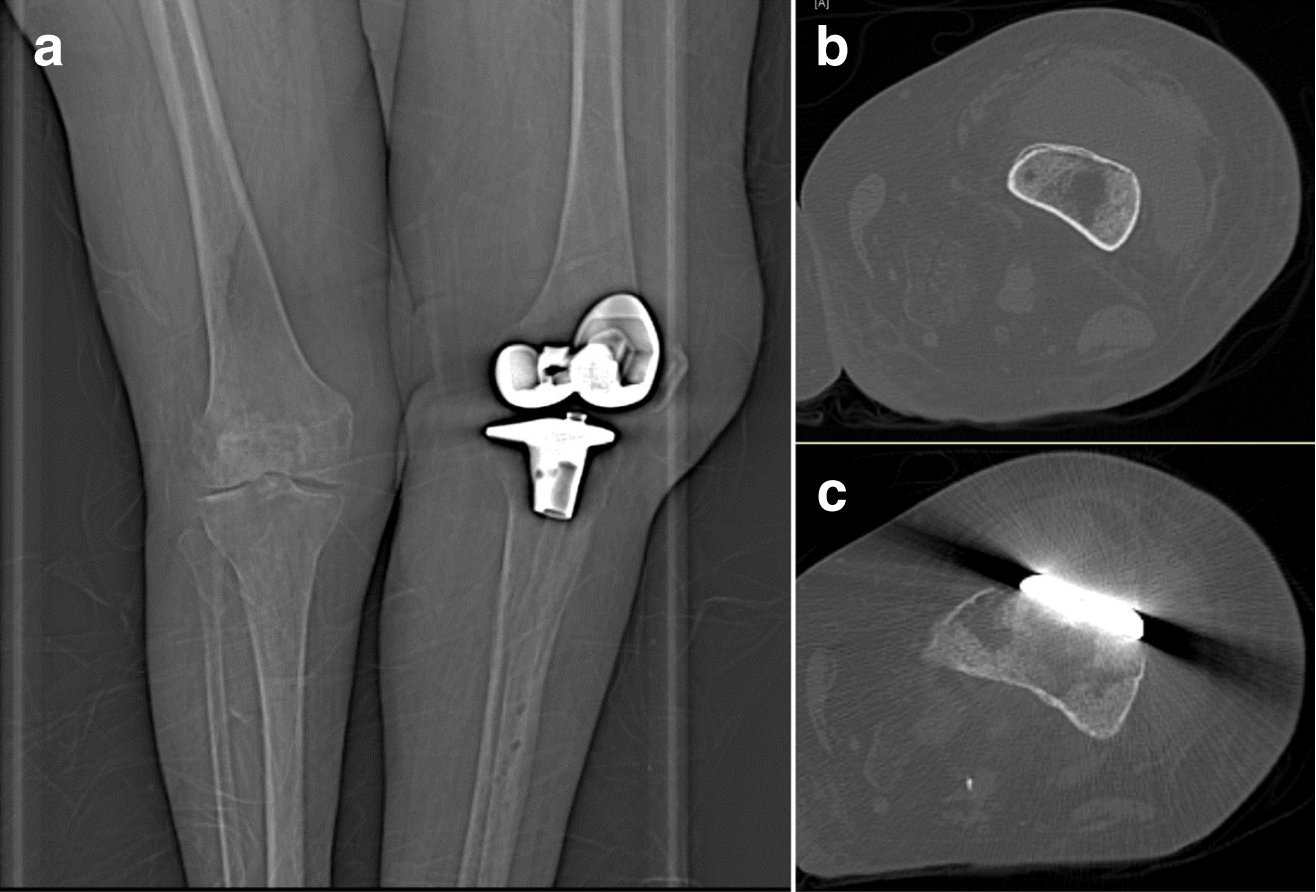
Frontal radiograph of the left knee at admission demonstrates normal post-operative appearance of prosthesis with no prosthetic loosening (**a**); Computer tomography (CT) shows an abscess in front of the knee prosthesis caused by *Pasteurella multocida* (**b** and **c**)

Five of our cases were associated with knee prostheses (Fig. 1) and one case with a hip prosthesis. Mean time delay between prosthetic joint implantation and infection onset was 7.6 years (±5.12 years, range 2–17). Local inflammation, which occurred in all six cases, was the most frequent clinical symptom, followed by pain in five cases, fever and swollen joints in four cases, and a fistula with purulent discharge inside the wound in two cases. Delay between first infectious signs and diagnosis of prosthetic joint infection was poorly reported in literature. Prosthesis loosening was observed in two cases including one case before infection and one case related to infection. None of the cases had positive blood culture of *P. multocida*.

Diagnoses of prosthetic joint infection caused by *P. multocida* were made by the positive culture of the percutaneous needle aspiration of articular fluid in two cases, and surgical deep samples in four cases. Surgical treatment was performed in six cases, including surgical lavage and debridement and prosthesis retention in three cases and prosthesis removal in three cases. All six cases received a combination of antibiotic treatment with amoxicillin and doxycycline. The mean time of antibiotic treatment was 8 months, range 6–18 months.

Six of our cases were in remission without apparent relapse at 3 years after end of treatment. One case was infected with *S. enteritidis* at 3 years after end of treatment on the same prosthesis.

## Discussion

We reported six cases of prosthetic joint infection caused by *P. multocida* managed in our centers over the last 20 years. The rate of prosthetic joint infection caused by *P. multocida* is low, representing thus only 0.1 % of all cases of prosthetic joint infection in our experience. To our knowledge, only 26 cases of prosthetic joint infection caused by *P. multocida* have been reported in the literature [13–35]. The mean age of cases reported was 67 years (±9.6 years, range 33–88 years). Prosthetic joint infections caused by *P. multocida* were usually a contiguous infection after scratching, biting or licking feet and more frequently localized in the knee than in the hip (21 cases vs. 5 cases) (Table 1). Haematogenous prosthetic joint infections caused by *P. multocida* were rarely reported and usually affected more than one prosthesis, such as two cases where both knee prostheses were infected after haematogenous dissemination [20, 21].

**Table 1:** Clinical characteristics and treatment of the 32 cases of prosthetic joint infection due to Pasteurella multocida including six cases in our study and the 26 cases reported in literature

Studies	References	Sex	Age (years)	Sites	Contacts with animal	Comorbidities	Medical treatment	Surgical treatment	Outcome
This report		Male	65	Knee	Dog licks	None	Amoxicillin, doxycycline	Surgical lavage and debridement	Cure
This report		Male	82	Hip	Cat scratches	None	Amoxicillin, doxycycline	Surgical lavage and debridement	Cure
This report		Female	63	Knee	Cat scratches	Diabetes mellitus	Amoxicillin, doxycycline	Replacement of prosthesis (two-stage exchange strategy)	Cure
This report		Male	65	Knee	Dog licks	Diabetes mellitus, Foot ulceration	Amoxicillin, doxycycline	Replacement of prosthesis (two-stage exchange strategy)	Cure
This report		Female	81	Knee	Cat contacts	Obesity (BMC at 55)	Amoxicillin, doxycycline	Replacement of prosthesis (two-stage exchange strategy)	Cure
This report		Female	85	Knee	Cat and dog contacts	None	Amoxicillin, doxycycline	Surgical lavage and debridement	Cure
Ferguson et al. (2014)	[13]	Female	67	Knee	Dog licks	None	Linezolid and ciprofloxacin	Surgical lavage and debridement	Cure
Romanò et al. (2013)	[35]	Female	82	Knee	Cat scratches	Rheumatoid arthritis	Amoxicillin-clavulanic acid, ciprofloxacin	Surgical lavage and debridement	Cure
Heydemann, Heydemann, and Antony (2010)	[34]	Male	66	Knee	Cat scratches	None	Ampicillin/sulbactam	Removal of tibia insert	Cure
Kadakia and Langkamer (2008)	[33]	Female	80	Knee	Cat bites	Breast carcinoma	Cefuroxime	Surgical lavage and debridement	Cure
Heym et al. (2006)	[32]	Female	72	Knee	Dog licks	None	Amoxicillin, doxycycline, ciprofloxacin, rifampin	Replacement of prosthesis (two-stage exchange strategy)	Cure
Mehta and Mackie (2004)	[30]	Female	84	Hip	Cat scratches	Rheumatoid arthritis	Benzyl penicillin, ciprofloxacin	Replacement of prosthesis (two-stage exchange strategy)	Cure
Mehta and Mackie (2004)	[30]	Female	57	Hip	Cat scratches	Rheumatoid arthritis	Flucloxacillin, benzyl penicillin	Replacement of prosthesis (two-stage exchange strategy)	Cure
Stiehl, Sterkin, and Brummitt (2004)	[29]	Male	63	Knee	Horse injury	None	Ciprofloxacin, piperacillin/tazobactam	Replacement of prosthesis (two-stage exchange strategy)	Cure
Polzhofer, Hassenpflug, and Petersen (2004)	[31]	Female	73	Knee	Cat bites	None	Ampicillin/sulbactam, clindamycin	Surgical lavage and debridement	Cure
Ciampolini, Timperley, and Morgan (2004)	[37]	Female	73	Knee	Cat scratches	None	Benzyl penicillin, ciprofloxacin	Replacement of prosthesis (two-stage exchange strategy)	Cure
Chikwe et al. (2000)	[28]	Male	69	Hip	Dog contacts	None	Information is not available	Replacement of prosthesis (two-stage exchange strategy)	Cure
Maradona et al. (1997)	[27]	Female	73	Knee	Dog bites	Diabetes mellitus	Penicillin, ciprofloxacin	Surgical lavage and debridement	Cure
Takwale et al. (1997)	[26]	Female	57	Hip	Cat scratches	Rheumatoid arthritis	Flucloxacillin, benzyl penicillin, metronidazole, ciprofloxacin	Replacement of prosthesis (two-stage exchange strategy)	Cure
Antuña et al. (1997)	[25]	Female	73	Knee	Dog bites	Rheumatoid arthritis	Ciprofloxacin	Surgical lavage and debridement	Cure
Gabuzda and Barnett (1992)	[22]	Female	88	Knee	Cat bites	None	Penicillin	Replacement of prosthesis (two-stage exchange strategy)	Cure
Guion and Sculco (1992)	[23]	Female	45	Knee	Dog scratches	Rheumatoid arthritis	Cefotaxime	Replacement of prosthesis (two-stage exchange strategy)	Cure
Braithwaite and Giddins (1992)	[24]	Female	48	Hip	Cat bites	Diabetes mellitus	Penicillin, Flucloxacillin	Replacement of prosthesis (two-stage exchange strategy)	Cure
Taillan et al. (1988)	[38]	Female	79	Knee	Cat bites	Rheumatoid arthritis, Acute leukemia	Pefloxacin	None	Cure
Orton and Fulcher (1984)	[21]	Female	74	Knee (both)	Cat bites	None	Ampicillin, penicillin, doxycycline	Replacement of prosthesis (two-stage exchange strategy)	Cure
Mellors and Schoen (1985)	[20]	Female	62	Knee (both)	Cat scratches	None	Penicillin	None	Cure
Gomez-Reino et al. (1980)	[19]	Female	64	Knee	Cat bites	None	Cephalothin	Replacement of prosthesis (two-stage exchange strategy)	Cure
Spagnuolo (1978)	[18]	Female	72	Knee	Cat bites	None	Penicillin	Surgical lavage and debridement	Cure
Arvan and Goldberg (1978)	[17]	Female	72	Knee	Cat bites	None	Penicillin	Surgical lavage and debridement	Cure
Sugarman, Quismorio, and Patzakis (1975)	[16]	Female	33	Knee	Dog licks	Rheumatoid arthritis	Cloxacillin, penicillin	Replacement of prosthesis (two-stage exchange strategy)	Cure
Griffin and Barber (1975)	[14]	Female	64	Knee	Cat scratches	Rheumatoid arthritis	Ampicillin	None	Cure
Maurer, Hasselbacher, and Schumacher (1975)	[15]	Female	55	Knee	Dog licks	Rheumatoid arthritis	Penicillin	None	Cure

Most of the cases in the literature occurred after animal bites or scratches, including 17 cases (65 %) after cat bites or scratches and eight cases (31 %) after dog scratches, bites, or licks and one case after a horse bite. Most of our cases involve animal contact or bites, consistent with the zoonotic origin of *Pasteurella multocida*.

Forty-two percent of the reported cases of prosthetic joint infection due to *P. multocida* presented at least one comorbidity. In general, diabetes mellitus appears to be a main comorbidity associated with prosthetic joint infections, but this underlying condition was identified in four cases only among the 32 reported cases (2 cases in the literature and 2 in this cases series). The major comorbidity related to prosthetic joint infection caused by *P. multocida* was rheumatoid arthritis treated with immunosuppressive drugs, which was observed in ten reported cases (38 %). None of our patients had rheumatoid arthritis and none was treated with immunosuppressive drugs, this observation illustrating that this factor is not perhaps as important as it might otherwise seem for prosthetic joint infection due to *P. multocida*.

Other uncommon comorbidities such as solid cancers have been observed in few reported cases, including one case of leukaemia and one case of breast carcinoma. None of our patients has a medical history of cancer during the management of prosthetic joint infections caused by *P. multocida*, but one of our patients developed breast cancer within 4 years after her prosthetic joint infection. Immunodeficiency should be considered a risk factor or comorbidity of prosthetic joint infection caused by *P. multocida,* especially in case of recurrence.

Prosthetic joint infections caused by *P. multocida* are monomicrobial infections and are sensitive to penicillin and doxycycline. Prosthesis removal remains a main treatment option in the cases reported in literature (14 cases; 54 %) followed by surgical debridement with prosthesis retention (8 cases; 31 %) and antibiotic treatment without surgery (4 cases; 15 %). The remission rate is high (85 %); only four reported cases treated initially with surgical debridement were subsequently treated with prosthesis removal [16, 19, 21, 32]. Three of the four cases had arthritis, one case was bacteremic to *P. multocida*, and he had a prosthetic joint infection due to *P. multocida* and *Pseudomonas aeruginosa*. We didn’t found any microbiologic information concerning the failure after medical treatment [21]. One case had first medical treatment 6 weeks and no surgical debridement [19]. One case had a cemented total knee arthroplasty with unchanged polyethylene tibial insert [32] (Table 1).

*P. multocida* appears to be a nonmotile coccobacillus recovered from the nasopharynx or gastrointestinal tract of wild animals, cats and dogs [6]. *P. multocida* are most often the result of direct tissue inoculation. We can assume that infections are locally contiguous and similar to the acute haematogenous prosthetic joint infection, surgical lavage, debridement and prosthesis retention associated with prolonged antimicrobial treatment should have a high success rate, which is the case in the literature and in our three cases.

*P. multocida* is known as a virulent pathogen, which has the ability to produce an in vitro biofilm [36]. Nevertheless, the case of *P. multocida* prosthetic joint infection can be caused by no-biofilm producer isolate [35]. We believe that increasing the studies on the biofilm role of *P. multocida* isolates in prosthetic joint infection should enable a better understanding of the pathogenesis of this bacterium and a better definition of treatment strategies. According to our findings and literature review, we believe that surgical lavage, debridement and prosthesis retention combined with prolonged antibiotic treatment is sufficient for the treatment of prosthetic joint infection caused by *P. multocida*.

Generally it is recommended that patients with animal bites receive systematic antibiotics to prevent infections due to *P. multocida* and other pathogens that form part of the oral animal flora [9]. Animal bites or scratches or licks from pets are a possibility in people with arthroplasty, particularly in the elderly, we think that they should be told of the risks and the action to be taken if it happens. Based on our review, we suggest that patients with orthopedic devices who have been bitten or scratched by animals should be early treated with either penicillin or doxycycline to avoid systemic spread and infection of the prosthesis with *P. multocida*.

## Conclusion

Prosthetic joint infections caused by *P. multocida* are rare and most commonly follow animal scratches or bites, but can occasionally occur after a short animal lick. Prosthetic joint infections caused by *P. multocida* were usually localized in the knee resulting from a contiguous infection, but haematogenous dissemination can occasionally affect more than one prosthesis. As there is no clear evidence that *P. multocida* could generate biofilm, we believe that early antibiotic therapy after surgical debridement could avoid prosthetic withdrawal, notably in elderly patients. However, early treatment after dog or cat scratch, bites or lick in a patient with joint prosthesis may be prevent prosthetic joint infection due to *Pasteurella multocida*.

## Abbreviations

CRIOA, Referral centre for the treatment of bone and joint infections “Centre de Référence des Infections Ostéo-Articulaires”; *P. multocida, Pasteurella multocida*

## References

[1] Lipsky BA, Berendt AR, Cornia PB, Pile JC, Peters EJG, Armstrong DG (2012). Executive summary: 2012 Infectious Diseases Society of America clinical practice guideline for the diagnosis and treatment of diabetic foot infections. Clin Infect Dis.

[2] Tena D, Romanillos O, Rodríguez-Zapata M, de la Torre B, Pérez-Pomata MT, Viana R (2007). Prosthetic hip infection due to Brucella melitensis: case report and literature review. Diagn Microbiol Infect Dis.

[3] Gupta A, Berbari EF, Osmon DR, Virk A (2014). Prosthetic joint infection due to Salmonella species: a case series. BMC Infect Dis.

[4] Vasoo S, Schwab JJ, Cunningham SA, Robinson TJ, Cass JR, Berbari EF (2014). Campylobacter prosthetic joint infection. J Clin Microbiol.

[5] Gomez E, Kennedy CC, Gottschalk M, Cunningham SA, Patel R, Virk A (2014). Streptococcus suis-related prosthetic joint infection and streptococcal toxic shock-like syndrome in a pig farmer in the United States. J Clin Microbiol.

[6] Hubbert WT, Rosen MN (1970). Pasteurella multocida infections. II. Pasteurella multocida infection in man unrelated to animal bite. Am J Public Health Nations Health.

[7] Griego RD, Rosen T, Orengo IF, Wolf JE (1995). Dog, cat, and human bites: a review. J Am Acad Dermatol.

[8] Luessenhop CP, Higgins LD, Brause BD, Ranawat CS (1996). Multiple prosthetic infections after total joint arthroplasty. Risk factor analysis. J Arthroplasty.

[9] Talan DA, Abrahamian FM, Moran GJ, Citron DM, Tan JO, Goldstein EJC (2003). Clinical presentation and bacteriologic analysis of infected human bites in patients presenting to emergency departments. Clin Infect Dis Off Publ Infect Dis Soc Am.

[10] Zimmerli W (2014). Clinical presentation and treatment of orthopaedic implant-associated infection. J Intern Med.

[11] Drancourt M, Carrieri P, Gévaudan M-J, Raoult D (2003). Blood agar and Mycobacterium tuberculosis: the end of a dogma. J Clin Microbiol.

[12] Drancourt M, Raoult D (2007). Cost-effectiveness of blood agar for isolation of mycobacteria. PLoS Negl Trop Dis.

[13] Ferguson KB, Bharadwaj R, MacDonald A, Syme B, Bal AM (2014). Pasteurella multocida infected total knee arthroplasty: a case report and review of the literature. Ann R Coll Surg Engl.

[14] Griffin AJ, Barber HM (1975). Letter: Joint infection by Pasteurella multocida. Lancet Lond Engl.

[15] Maurer KH, Hasselbacher P, Schumacher HR (1975). Letter: Joint infection by Pasteurella multocida. Lancet Lond Engl.

[16] Sugarman M, Quismorio FP, Patzakis MJ (1975). Letter: Joint infection by Pasteurella multocida. Lancet Lond Engl.

[17] Arvan GD, Goldberg V (1978). A case report of total knee arthroplasty infected by Pasteurella multocida. Clin Orthop.

[18] Spagnuolo PJ (1978). Pasteurella multocida infectious arthritis. Am J Med Sci.

[19] Gomez-Reino JJ, Shah M, Gorevic P, Lusskin R. Pasteurella multocida arthritis. Case report. J Bone Jt Surg. 1980;62:1212–3. 7430213

[20] Mellors JW, Schoen RT (1985). Pasteurella multocida prosthetic joint infection. Ann Emerg Med.

[21] Orton DW, Fulcher WH (1984). Pasteurella multocida: bilateral septic knee joint prostheses from a distant cat bite. Ann Emerg Med.

[22] Gabuzda GM, Barnett PR (1992). Pasteurella infection in a total knee arthroplasty. Orthop Rev.

[23] Guion TL, Sculco TP (1992). Pasteurella multocida infection in total knee arthroplasty. Case report and literature review. J Arthroplasty.

[24] Braithwaite BD, Giddins G (1992). Pasteurella multocida infection of a total hip arthroplasty. A case report. J Arthroplasty.

[25] Antuña SA, Méndez JG, Castellanos JL, Jimenez JP (1997). Late infection after total knee arthroplasty caused by Pasteurella multocida. Acta Orthop Belg.

[26] Takwale VJ, Wright ED, Bates J, Edge AJ (1997). Pasteurella multocida infection of a total hip arthroplasty following cat scratch. J Infect.

[27] Maradona JA, Asensi V, Carton JA, Rodriguez Guardado A, Lizón CJ (1997). Prosthetic joint infection by Pasteurella multocida. Eur J Clin Microbiol Infect Dis.

[28] Chikwe J, Bowditch M, Villar RN, Bedford AF (2000). Sleeping with the enemy: Pasteurella multocida infection of a hip replacement. J R Soc Med.

[29] Stiehl JB, Sterkin LA, Brummitt CF (2004). Acute pasteurella multocida in total knee arthroplasty. J Arthroplasty.

[30] Mehta H, Mackie I (2004). Prosthetic joint infection with Pasturella multocida following cat scratch: a report of 2 cases. J Arthroplasty.

[31] Polzhofer GK, Hassenpflug J, Petersen W (2004). Arthroscopic treatment of septic arthritis in a patient with posterior stabilized total knee arthroplasty. Arthroscopy.

[32] Heym B, Jouve F, Lemoal M, Veil-Picard A, Lortat-Jacob A, Nicolas-Chanoine MH (2006). Pasteurella multocida infection of a total knee arthroplasty after a “dog lick”. Knee Surg Sports Traumatol Arthrosc.

[33] Kadakia AP, Langkamer VG (2008). Sepsis of total knee arthroplasty after domestic cat bite: should we warn patients?. Am J Orthop Belle Mead NJ.

[34] Heydemann J, Heydemann JS, Antony S (2010). Acute infection of a total knee arthroplasty caused by Pasteurella multocida: a case report and a comprehensive review of the literature in the last 10 years. Int J Infect Dis.

[35] Romanò CL, De Vecchi E, Vassena C, Manzi G, Drago L (2013). A case of a late and atypical knee prosthetic infection by no-biofilm producer Pasteurella multocida strain identified by pyrosequencing. Pol J Microbiol.

[36] Olson ME, Ceri H, Morck DW, Buret AG, Read RR (2002). Biofilm bacteria: formation and comparative susceptibility to antibiotics. Can J Vet Res.

[37] Ciampolini J, Timperley J, Morgan M (2004). Prosthetic joint infection by cat scratch. J R Soc Med.

[38] Taillan B, Jullien JP, Fuzibet JG, Dujardin P, Bernard E, Gagnerie F (1988). Septic arthritis due to Pasteurella multocida. 3 new case reports. Rev Rhum Mal Osteoartic.

